# Increased Serum Level of Cyclopropaneoctanoic Acid 2-Hexyl in Patients with Hypertriglyceridemia-Related Disorders

**DOI:** 10.1007/s11745-016-4141-1

**Published:** 2016-03-22

**Authors:** Adriana Mika, Piotr Stepnowski, Michal Chmielewski, Sylwia Malgorzewicz, Lukasz Kaska, Monika Proczko, Krzysztof Ratnicki-Sklucki, Maciej Sledzinski, Tomasz Sledzinski

**Affiliations:** Department of Environmental Analysis, Faculty of Chemistry, University of Gdansk, 63 Wita Stwosza Str., 80-308 Gdansk, Poland; Department of Nephrology, Transplantology and Internal Medicine, Medical University of Gdansk, 7 Debinki Str., 80-952 Gdansk, Poland; Department of Clinical Nutrition, Medical University of Gdansk, 7 Debinki Str., 80-952 Gdansk, Poland; Department of General, Endocrine and Transplant Surgery, Medical University of Gdansk, 17 Smoluchowskiego Str., 80-214 Gdansk, Poland; Department of Emergency Medicine, Medical University of Gdansk, 17 Smoluchowskiego Str., 80-214 Gdansk, Poland; Department of Pharmaceutical Biochemistry, Medical University of Gdansk, 1 Dębinki Str., 80-211 Gdansk, Poland

**Keywords:** Cyclopropaneoctanoic acid 2-hexyl, Obesity, Chronic kidney disease, Hypertriglyceridemia

## Abstract

We recently reported the presence of various cyclopropane fatty acids—among them, cyclopropaneoctanoic acid 2-hexyl—in the adipose tissue of obese women. The aim of this study was to verify whether the presence of cyclopropaneoctanoic acid 2-hexyl in human serum was associated with obesity or chronic kidney disease (both being related to dyslipidemia), and to find potential associations between the serum level of this compound and specific markers of the these conditions. The serum concentration of cyclopropaneoctanoic acid 2-hexyl was determined by gas chromatography–mass spectrometry (GC–MS) in non-obese controls, obese patients, obese patients after a 3-month low-calorie diet, and individuals with chronic kidney disease. Obese patients and those with chronic kidney disease presented with higher serum levels of cyclopropaneoctanoic acid 2-hexyl than controls. Switching obese individuals to a low-calorie (low-lipid) diet resulted in a reduction in this fatty acid concentration to the level observed in controls. Cyclopropaneoctanoic acid 2-hexyl was also found in foods derived from animal fat. Serum concentrations of triacylglycerols in the analyzed groups followed a pattern similar to that for serum cyclopropaneoctanoic acid 2-hexyl, and these variables were positively correlated with each other among the studied groups. Patients with hypertriglyceridemia-related conditions presented with elevated serum levels of cyclopropaneoctanoic acid 2-hexyl. Our findings suggest that its high serum level is related to high serum triacylglycerol concentrations rather than to body mass or BMI.

## Introduction

Cyclopropane fatty acids (CFA) are found in phospholipids and glycolipids of cell membranes from many bacterial species, and likely play a role in the pathogenesis of bacterial infections [1]. These fatty acids (FA) have also been found in certain eukaryotes, including protozoa [2] and plants [3, 4]. However, few studies have documented the presence of CFA in animals [5–7]. We recently found four CFA—cyclopropaneoctanoic acid 2-hexyl, cyclopropaneoctanoic acid 2-octyl, cyclopropanenonanoic acid and 2-[[2-[(2-ethylcyclopropyl)methyl]cyclopropyl]methyl] acid—in the adipose tissue of obese women [8]. Cyclopropaneoctanoic acid 2-hexyl (CPOA2H), also referred to as 9,10-methylene hexadecanoic acid, was the most abundant CFA, as well as the only CFA detectable in their serum [8]. The results of previous studies suggest that CFA may play an important role in the human body, given their regulatory properties such as control of cyclooxygenase activity [9], actomyosin ATPase [10], protein kinase C-ɛ [11], stearoyl-CoA desaturase [12] and inflammation [13]. CFA present in bacteria and plants are synthesized from unsaturated FA due to involvement of cyclopropane synthase, an enzyme catalyzing the addition of the methylene group from *S*-adenosylmethionine to the double bond of FA precursors [1, 14]. To date, however, this enzyme has not been identified in animals. Since our previous research [8] included adipose tissue and serum from obese women, we thought that it would be interesting to examine whether the presence of CFA was obesity-specific. To this end, we determined serum levels of CPOA2H in non-obese controls, obese patients, obese persons after a 3-month low-calorie diet and individuals with chronic kidney disease (CKD), i.e. with a disease related to dyslipidemia. We also looked for potential associations between the levels of CPOA2H, serum concentrations of lipids, and other biochemical and anthropometrical parameters of the study subjects.

## Materials and Methods

### Patients

The study included 76 women. Ten obese patients were examined at an ambulatory surgical facility as a part of the qualification for bariatric surgery performed at the Department of General, Endocrine and Transplant Surgery, Medical University of Gdansk (Poland). Another 29 obese patients were on a low-calorie diet (1000–1200 kcal/day, high protein and low fat and carbohydrate content) for 3 months prior to the study. The mean weight loss in this group was 9.75 kg. Patients with CKD are another population in whom dyslipidemia is a common finding. Although the etiology of lipid disorders observed in individuals with CKD is different from that in obese patients, the major disturbances, including elevated triacylglycerol concentration, are essentially similar . In addition, like obese subjects, patients with CKD present with clearly increased cardiovascular risk. Consequently, 15 women were selected from a pool of patients who had been subjected to peritoneal dialysis due to end-stage renal failure at the Department of Nephrology, Transplantology and Internal Medicine, Medical University of Gdansk. The control group included 22 healthy non-obese volunteers who were referred for an annual health check-up. The patients' serum was obtained retrospectively from a sample bank, and clinical and demographic characteristics were extracted from a clinical database. The protocol of the study was approved by the local bioethics committee at the Medical University of Gdańsk (protocol nos. NKEBN/475/2012 and NKEBN/614/2013-2014), and patients gave their informed consent to use of their clinical data and analyses performed in the study. The study protocol adhered to the tenets of the Declaration of Helsinki of the World Medical Association. Characteristics of the study participants are presented in Table 1. A Tanita scale was used to estimate the parameters of body constitution, including body weight, body height and BMI. BMI was calculated from the following formula: BMI (kg/m^2^) = body weight (kg)/body height (m)^2^. Blood samples for determining basic laboratory parameters, such as serum albumin, C-reactive protein, total cholesterol, and triacylglycerols, were collected from patients after an overnight fast. All laboratory parameters were determined at the Central Clinical Laboratory, Medical University of Gdansk. The remaining aliquots of serum were stored at −80 °C for fatty acid composition analysis.

**Table 1:** Clinical characteristics of the study participants

Group	Controls	Obese patients	Obese patients after diet	Patients with chronic kidney disease
Number of subjects included	22	10	29	15
Inclusion criteria	BMI < 27 kg/m^2^ Age 18–65 years No clinical evidence of endocrine (including diabetes), cardiac, hepatic, mental, neoplastic or renal disease	BMI > 35 kg/m^2^ Age 18–65 years No clinical evidence of endocrine (including diabetes), cardiac, hepatic, mental, neoplastic or renal disease	BMI > 35 kg/m^2^ before dietary intervention Age 18–65 years >5-kg weight loss No clinical evidence of endocrine (including diabetes), cardiac, hepatic, mental, neoplastic or renal diseases	Treatment by peritoneal dialysis for end-stage renal failure No clinical evidence of endocrine (including diabetes), cardiac, hepatic, mental or neoplastic diseases
Dietary intervention	No dietary recommendation	No dietary recommendation	Low-calorie diet (1000–1200 kcal/day) for 3 months prior to blood collection. The patients were advised to remain on a high-protein, low-fat and low-carbohydrate diet (lean meat, eggs, fish, cottage cheese, yogurt, vegetables, grains, corn cereals, sponge cake, biscuits) and to avoid products containing sucrose	Normal-calorie diet recommended for dialyzed patients. The patients were advised to reduce potassium and phosphate intake. Protein intake was set at 1.2 g/kg/day

### Gas Chromatography–Mass Spectrometry (GC–MS) Analysis of FA

Total lipids were extracted from patient serum and from cow's milk, porcine, chicken, trout fat, and rapeseed oil using the method described by Folch *et al*. [21]. Subsequently, FA were derivatized to FA methyl esters (FAME) using 10 % boron trifluoride-methanol solution. FA profiles in the adipose tissue and serum lipids were analyzed with the GCMS QP-2010SE unit (Shimadzu, Kyoto, Japan), as described previously [8]. In our recent study, we confirmed the identification of CPOA2H by derivatization of FA to picolinyl esters, followed by GC–MS analysis [8]. The presence of this FA in serum was further confirmed using a CPOA2H standard obtained from Matreya, LLC (State College, PA, USA). Concentrations of individual FA, including CPOA2H, were calculated based on the amount of added internal standard and volume of serum used for the procedure.

### Statistical Analysis

The statistical significance of intergroup differences was determined with one-way analysis of variance (ANOVA), and the Tukey post hoc test was used for multiple comparisons. Inter-group differences were considered significant at *p* < 0.05. All data are presented as means ± standard errors of the mean (SEM). Normality was verified with the Shapiro–Wilk test. The relationships between pairs of variables were determined on the basis of linear regression analysis. All calculations were conducted using SigmaPlot for Windows, version 11.0 (2008; Systat Software Inc).

## Results

**Fig. 1:**
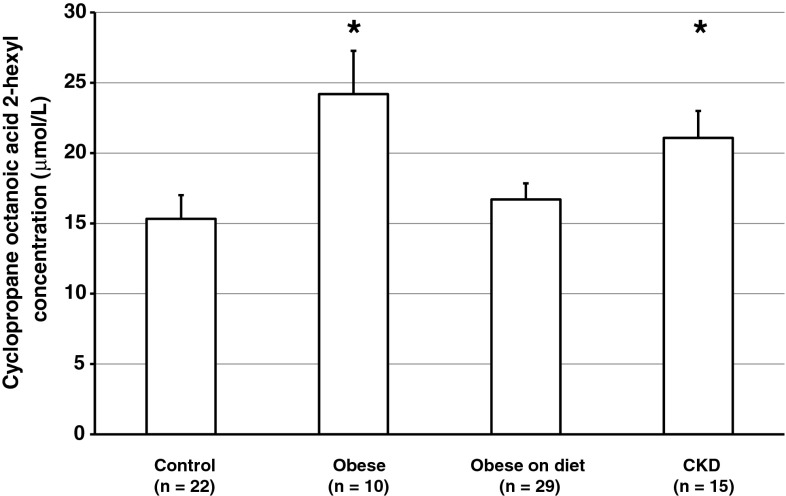
Serum concentrations of cyclopropaneoctanoic acid 2-hexyl in study subjects, including non-obese controls, obese patients, obese subjects after a 3-month low-calorie (low-lipid) diet and individuals with chronic kidney disease (CKD). Data are presented as mean ± SEM. *p < 0.05 compared to the controls

Obese subjects and CKD patients differed from the controls in terms of whole serum lipids FA concentrations. Obese women presented with higher serum concentrations of saturated FA (SFA) and monounsaturated FA (MUFA) (Table 2). Obese women after a low-calorie diet did not differ significantly from controls in terms of fatty acid concentrations (Table 2). Patients with chronic kidney disease presented with lower n-6 PUFA levels than the controls (Table 2). Serum concentrations of CPOA2H were significantly higher in morbidly obese women than in non-obese subjects (24.2 ± 3.07 vs. 15.3 ± 1.69 µmol/L, *p* < 0.05; Fig. 1). Serum levels of CPOA2H in obese women after a low-calorie diet were similar to those found in the controls (16.7 ± 1.15 vs 15.3 ± 1.69 µmol/L, Fig. 1). CKD patients presented with significantly higher serum concentrations of CPOA2H than did controls (21.1 ± 1.92 vs 15.3 ± 1.69 μmol/L, *p* < 0.05; Fig. 1).

**Table 2:** Fatty acid concentrations in the whole serum lipids of the study subjects

Fatty acid	Controls (μmol/L)	Obese patients (μmol/L)	Obese patients after diet (μmol/L)	Patients with chronic kidney disease (μmol/L)
12:0	15.1 ± 1.54	24.2 ± 6.49	16.4 ± 1.68	15.7 ± 1.75
14:0	93.5 ± 9.04	188 ± 59.2^#^	94.1 ± 7.29	115 ± 11.9
15:0	26.1 ± 2.21	31.7 ± 6.03	25.8 ± 1.71	26.8 ± 2.28
16:0	1569 ± 125	2393 ± 482^#^	1820 ± 92.7	1697 ± 156
17:0	23.4 ± 1.78	29.7 ± 5.91^#^	21.7 ± 1.02	28.8 ± 2.68^#^
18:0	525 ± 42.2	668 ± 135	491 ± 24.7	584 ± 69.2
19:0	3.48 ± 0.32	3.73 ± 0.78	3.08 ± 0.25	3.13 ± 0.37
20:0	11 ± 0.84	15.8 ± 3.90	11.2 ± 0.64	13.4 ± 1.40
21:0	3.78 ± 0.34	3.65 ± 0.46	4.31 ± 0.31	3.06 ± 0.29
22:0	17.4 ± 1.12	19.6 ± 3.37	18.2 ± 1.02	16.3 ± 1.58
23:0	6.77 ± 0.54	7.38 ± 1.48	6.57 ± 0.33	6.25 ± 0.85
24:0	16.8 ± 1.09	18.1 ± 2.92	17.1 ± 0.93	14.6 ± 1.24
SFA	2311 ± 14.9	3403 ± 57.7^#^	2530 ± 10.3	2525 ± 19.7
14:1	4.74 ± 0.66	11.6 ± 4.6^#^	4.76 ± 0.46	6.26 ± 0.81
16:1	206 ± 21.7	386 ± 74.9*	267 ± 22.1	265 ± 30.1
18:1	1578 ± 132	2445 ± 470^#^	1869 ± 107	1959 ± 223
20:1	12.0 ± 1.21	19.0 ± 4.59	13.8 ± 1.06	19.5 ± 1.83*
24:1	14.2 ± 1.49	19.6 ± 3.58	14.2 ± 0.74	14.2 ± 1.43
MUFA	1815 ± 31.1	2880 ± 110^#^	2169 ± 25.3	2263 ± 50.5
18:2n-6	1742 ± 127	1778 ± 2370	1532 ± 91.6	1257 ± 107^#^
20:4n-6	376 ± 40.1	439 ± 71.9	430 ± 22.1	230.85 ± 19.8^#^
20:3n-6	82.7 ± 8.01	137 ± 18.2*	92.5 ± 7.39	67.2 ± 7.24
20:2n-6	16.1 ± 2.80	17.0 ± 1.89	12.8 ± 0.97	15.2 ± 1.39
22:5n-6	6.26 ± 0.66	8.14 ± 1.32	6.94 ± 0.46	4.23 ± 0.41^#^
22:4n-6	10.7 ± 1.65	13.8 ± 2.23	10.3 ± 0.65	4.88 ± 0.59^#^
PUFAn-6	2234 ± 28.9	2393 ± 53.8	2084 ± 19.1	1579 ± 22.1^#^
18:3n-3	19.2 ± 2.26	42.5 ± 10.9*	21.9 ± 2.41	15.3 ± 1.70
20:5n-3	59.1 ± 7.34	72.2 ± 19.2	60.0 ± 6.04	53.5 ± 5.09
20:4n-3	8.02 ± 1.08	11.6 ± 1.71	6.78 ± 0.79	8.80 ± 0.96
22:6n-3	96.0 ± 13.1	83.7 ± 11.7	106 ± 7.95	71.5 ± 6.60
22:5n-3	26.5 ± 2.91	34.0 ± 5.65	25.2 ± 1.63	20.6 ± 1.58
PUFAn-3	209 ± 5.02	244 ± 9.20	220 ± 3.17	170 ± 2.42

Data are presented as mean ± SEM

* *p* < 0.01; ^#^ *p* < 0.05 indicates a statistically significant difference compared to controls

To identify the potential pathophysiological role of CPOA2H serum concentration, we also compared study groups in terms of selected anthropometric and biochemical parameters (Table 3). Since CPOA2H was originally found in serum from obese women [8], we first analyzed the BMI of our subjects. Both serum CPOA2H and BMI were significantly higher in obese women than in controls. However, while the BMI of obese women who had been maintaining a low-lipid diet was only about 3.5 % lower than prior to diet implementation, their serum CPOA2H concentrations were similar to those found in non-obese controls, implying that CPOA2H found in our subjects might originate from food. To verify this hypothesis, we determined the content of this FA in selected high-fat foods. CPOA2H was found in cow's milk, porcine, chicken and trout fat, but not in rapeseed oil (Table 4). CKD patients, whose BMI was similar to that of the controls, presented with significantly higher CPOA2H levels. As shown in Table 3, serum concentrations of triacylglycerols (TAG) followed a pattern similar to serum CPOA2H levels. We conducted linear regression analysis to determine the exact relationship between CPOA2H and various anthropometric and laboratory parameters including age, BMI, albumin, CRP, total cholesterol and TAG concentrations among the studied groups of patients. We found strong positive correlations between CPOA2H and serum TAG in obese subjects and controls (Table 5). We also found positive correlations between CPOA2H and serum cholesterol in both groups of obese patients (Table 5).

**Table 3:** Selected characteristics of the study participants

	Controls	Obese patients	Obese patients after diet	Patients with chronic kidney disease
Age (years)	35.6 ± 2.62	46.4 ± 3.52	40.6 ± 1.75	55.1 ± 3.74*
BMI (kg/m^2^)	22.6 ± 0.67	43.3 ± 1.88*	40.7 ± 1.01*	26.5 ± 1.35
Albumin (g/L)	41.4 ± 0.74	40.0 ± 1.37	37.0 ± 0.93	39.6 ± 1.38
CRP (mg/L)	0.75 ± 0.16	11.8 ± 3.21*	5.71 ± 0.79*	4.72 ± 0.88*
Total cholesterol (mg/dL)	183 ± 5.42	188 ± 13.4	184 ± 7.92	227 ± 10.1*
Triacylglycerols (mg/dL)	72.8 ± 9.06	180 ± 24.1*	122 ± 8.20*	145 ± 17.8*

Data are presented as mean ± SEM

* *p* < 0.01 indicates statistically significant difference compared to controls

**Table 4:** Cyclopropaneoctanoic acid 2-hexyl content in commonly consumed high-fat foods

Food	Porcine fat (μg/g)	Chicken fat (μg/g)	Trout fat (μg/g)	Cow's milk (μmol/L)	Rapeseed oil
Cyclopropaneoctanoic acid 2-hexyl content	12.6	5.53	3.01	19.6	nd

**Table 5:** Correlation coefficients between serum concentrations of cyclopropaneoctanoic acid 2-hexyl and selected biochemical and anthropometric parameters

	Controls	Obese patients	Obese patients after diet	Patients with chronic kidney disease
Age	0.40	0.37	0.18	0.47
BMI	0.28	−0.30	−0.01	0.16
Albumin	−0.01	0.47	0.16	0.16
CRP	−0.01	−0.36	0.05	0.06
Total cholesterol	0.29	0.71*	0.47*	0.01
Triacylglycerols	0.66*	0.72*	0.71*	0.40

* *p* < 0.01 indicates statistical significance

## Discussion

The FA profile in human serum is an established determinant of metabolic and cardiovascular risk [15]. A recent identification of CFA in human adipose tissue and blood [8] stimulated questions about their physiological role. We recently found CPOA2H in the serum of patients with obesity, a disease associated with dyslipidemia, inflammation and increased cardiovascular risk. Consequently, we decided to compare the serum concentrations of this FA in obese subjects and non-obese controls, and searched for associations between this parameter and selected markers of metabolic disorders. In addition, our study included individuals with CKD, a disease which is also associated with various lipid disorders and increased cardiovascular risk [16], but usually not with excess body weight. Obese women presented with higher CPOA2H levels than non-obese controls. However, excess body weight did not seem to be a principal determinant of the serum concentration of this FA: although obese patients who had been subjected to a 3-month low-calorie diet still differed from non-obese controls in terms of BMI, they showed similar CPOA2H concentrations. In contrast, patients with CKD showed no significant difference from controls in terms of BMI, despite significantly higher serum CPOA2H levels. Lastly, we found no significant correlation between serum CPOA2H and BMI.

Since statistically significant correlations were found between serum CPOA2H and TAG levels, one can speculate that CPOA2H interacts with the endogenous synthesis of lipids—for instance, by influencing transcription factors involved in this process, as reported previously for other FA [17–19]. However, this hypothesis must be verified empirically, for example, by treating lipogenic cells with this FA. Importantly, the correlation between serum levels of CPOA2H and TAG may also reflect dietary intake of this FA with high-fat foods. Caligiani *et al*. recently reported the presence of an 18-carbon CFA in cow's milk and dairy products [20]. In our previous study, we found CPOA2H in the TAG fraction of human blood [8], which suggests that this FA may originate from chylomicrons transporting ingested lipids. This hypothesis is also supported by the fact that obese individuals subjected to a 3-month low-calorie (low-lipid) diet presented with significantly lower serum levels of CPOA2H. Furthermore, the findings presented here are consistent with the results of our previous study [8], in which we found decreased CPOA2H levels in the adipose tissue of rats that had been provided 50 % of the total amount of food consumed by the controls for a period of 1 month. The fact that this FA has been found in certain high-fat foods commonly consumed in our country also supports this hypothesis, although we should note that the dietary origin of CPOA2H in the serum of patients is speculation on our part. Interestingly, patients with CKD—albeit without obesity—presented with high concentrations of CPOA2H. The diet recommended in CKD, however, is not rich in fat (Table 1). Therefore, high serum concentrations of CPOA2H in CKD patients seem to be associated rather with increased serum levels of TAG.

The relevance of altered serum concentrations of CPOAH2 remains unclear. Given the positive correlations between concentrations of this FA, TAG and cholesterol, as well as the elevated levels found in both obese individuals and patients with kidney disease, we can speculate that CPOAH2 negatively affect the cellular metabolism of lipids. However, elevated levels of CPOA2H in these groups of patients may also represent an adaptive response. In vitro studies of hepatocytes and adipose tissue cells are needed to answer this question.

## Conclusions

This study showed that hypertriglyceridemia observed during the course of diseases such as CKD and obesity is associated with an increase in serum concentration of CPOA2H. Future studies should elucidate whether this uncommon FA may influence cellular metabolism in humans.

## Abbreviations

BMI: Body mass index
GC–MS: Gas chromatography–mass spectrometry
CFA: Cyclopropane fatty acid
FA: Fatty acid
CPOA2H: Cyclopropaneoctanoic acid 2-hexyl
SFA: Saturated fatty acid
MUFA: Monounsaturated fatty acid
PUFA: Polyunsaturated fatty acid
CKD: Chronic kidney disease
FAME: Fatty acid methyl ester
SEM: Standard error of the mean
TAG: Triacylglycerol
